# Depressive symptoms in women with premature ovarian insufficiency (POI): a cross-sectional observational study

**DOI:** 10.1097/GME.0000000000002614

**Published:** 2025-07-15

**Authors:** Charissa van Zwol‐Janssens, Yvonne V. Louwers, Joop S.E. Laven, Jits Schipper, Geranne Jiskoot

**Affiliations:** Department of Obstetrics and Gynecology, Division of Reproductive Endocrinology and Infertility, Erasmus MC, Rotterdam, The Netherlands

**Keywords:** Depressive symptoms, Menopause, Premature ovarian insufficiency

## Abstract

**Objective:**

To explore associations between clinical and patient-reported variables and depressive symptoms in women with premature ovarian insufficiency (POI).

**Methods:**

An exploratory cross-sectional observational study was conducted using data from the center of expertise for women with POI, Erasmus MC, the Netherlands. To identify variables associated with depressive symptoms, as assessed by patient and clinician-reported outcome measures, we used logistic regression models.

**Results:**

Between April 2020 and December 2023, 345 women with POI were included. In this cohort, the prevalence of depressive symptoms was 29.9%. No significant difference was found in depressive symptoms between women using estrogen plus progestogen therapy (EPT) (41.7%) and those not using EPT (42.6%, *P*=0.89). Younger age at diagnosis (*P*=0.01), POI due to a genetic cause (*P*=0.04), severe menopausal symptoms (*P*<0.001), and lack of emotional support (*P*<0.001) were independently associated with depressive symptoms. The use of EPT or levels of estradiol were not associated with depressive symptoms.

**Conclusions:**

The high prevalence of depressive symptoms among women with POI underscores a need for targeted mental health support. Our findings highlight that younger age at diagnosis, severe menopausal symptoms, and fertility-related grief are associated with depression in this population. Given that estradiol levels did not correlate with depressive symptoms, this suggests that psychosocial factors are crucial. Psychological interventions should focus on these factors to address the unique needs of this population.

Premature ovarian insufficiency (POI), also known as premature menopause or in former days as premature ovarian failure, is a condition in which the ovaries cease to function normally before the age of 40.^1^ POI is a life-changing diagnosis with profound physical, psychological, and social consequences.^2^ Women with POI not only experience the symptoms associated with estrogen deficiency but also experience the loss of reproductive function. A combination of these factors may lead to various psychological problems that significantly impact their quality of life (QoL).

POI is linked to an elevated lifetime risk for depression and anxiety. A recent meta-analysis revealed an odds ratio (OR) of 3.3 for depression and 4.9 for anxiety in women with POI compared with those without the condition.^3^ While the precise mechanisms underlying anxiety and depression in POI are yet to be fully elucidated, potential contributing factors may involve both neuroendocrine dysregulation and psychosocial stressors. Both a large increase in FSH levels^4^ and low levels of estradiol (E2)^5^ are associated with an increased risk of anxiety and depression. On the psychosocial front, the experience of infertility and the additional burdens imposed by estrogen deficiency, such as vasomotor symptoms, reduced short term memory function, reduced bone mineral density, and an increased risk of cardiovascular disease,^6–8^ may contribute to the heightened risk of anxiety and depression in women with POI.

Previous research has shown a vulnerability for depressive symptoms during the menopause transition.^9^ The incidence of depressive symptoms is 2-3 times higher in perimenopausal women compared with premenopausal women. Therefore, there seems to be a relation between depressive symptoms and menopausal status. This study suggested that hot flushes and sleep disruption caused by hot flushes are especially related to depressive symptoms. Also, women who received hormone therapy (HT) seem to have a lower risk of developing depressive symptoms compared with those who did not use HT.^9^

It would be helpful to identify factors that are associated with developing depression in women with POI. A recent study by Handing et al^10^ employed machine learning techniques to identify the most significant factors associated with depression risk in middle-aged European women. The findings revealed that social isolation, poor self-rated health, and family burden emerged as the primary determinants of depression susceptibility. We hypothesized that similar factors are associated with depression in women with POI. To further our understanding of the factors that contribute to depression in women with POI, we propose a large-scale cross-sectional study to identify variables that are associated with depression in this population.

## METHODS

We performed a cross-sectional study to identify variables that are associated with depressive symptoms in women with POI using clinician and patient-reported outcome measures (PROM). The study was conducted in accordance with the Declaration of Helsinki and approved by the Ethics Committee of Erasmus Medical Centre (The Netherlands) (protocol code MEC-2023-0094; May 10, 2023). Informed consent was obtained from all women involved in the study.

### Participants

All women with POI who attended our multidisciplinary POI care unit between April 2020 and December 2023 for the first time were included. Women who did not meet the criteria for POI or did not complete the questionnaires were excluded. POI was diagnosed according to the ESHRE 2016 guideline, women should have oligo/amenorrhea for at least 4 months, and an elevated FSH level (>25 IU/L) on 2 occasions at least 4 weeks apart.^1^

### Patient-reported outcome measures

The method of collecting PROMs at our multidisciplinary POI care unit has been described before.^11^ In short, before the first visit, women were asked to complete multiple PROM questionnaires: Beck Depression Inventory (BDI-II), Greene Climacteric scale (GCS), and in case of grief related to infertility, the Fertility Quality of Life tool (FertiQoL). In addition, a questionnaire about general health, medical history and lifestyle, and a questionnaire about emotional support (PROMIS) were conducted. Depressive symptoms were classified by a total BDI-II score above 19.^12^

### Clinician-reported outcome measures

During the first visit to our multidisciplinary POI care unit, a comprehensive patient assessment and personalized health care plan was formulated. This assessment typically encompasses the measurement of vital signs, including blood pressure, weight, and height, to calculate body mass index (BMI). A comprehensive blood panel was conducted to evaluate hormonal levels, lipid profile, and thyroid function (assessed by thyroid stimulating hormone [TSH]). To investigate neuroendocrine dysregulation, luteinizing hormone (LH), follicle-stimulating hormone (FSH), estradiol, and progesterone were included in the analysis. In addition, anti-Müllerian hormone was analyzed as a proxy for ovarian reserve, and vitamin D levels were included due to their association with depression.

### Statistical analysis

Based on a literature review and predefined hypotheses, potential variables associated with depressive symptoms were selected. To account for uncertainty in variable inclusion, all available variables were initially examined using univariate logistic regression models. Variables with a *P*-value <0.20 were then included in a multivariate model. All variables were standardized for interpretability. Categorical variables included a missing category to accommodate missing data in clinical practice. We used country of birth as a proxy for ethnicity, with the Netherlands serving as the reference group. Similarly, being single was used as the reference category for relationship status. In a multivariate backwards elimination procedure, variables that did not (significantly, *P*<0.05) contribute to the dependent measure were removed from the model one by one. Four sub-analyses were conducted: (1) among women who expressed a desire to conceive, (2) young women aged under 30, (3) among women with idiopathic POI, and (4) to identify specific domains and questions in the GCS, FertiQol, and PROMIS for emotional support that were significantly associated with depressive symptoms. Given the exploratory nature of this study, we have not applied formal corrections for multiple testing. The statistical analyses were performed using the Statistical Package of Social Sciences version 24.0 for Windows (SPSS Inc., Chicago, IL).

## RESULTS

Between April 2020 and December 2023, 345 women with POI attended our multidisciplinary POI care unit. Baseline characteristics of these women are shown in Table 1. Of the 345 women, 103 (29.9%) reported depressive symptoms, while 242 (70.1%) did not. In this cohort, a considerable number of women (42.3%) were already using estrogen plus progestogen therapy (EPT) at baseline. However, there was no difference in depressive symptoms between women using EPT and those not using EPT (*P*=0.89).

**TABLE 1:** Baseline characteristics of women with POI

	Women with POI (N = 345)	Women with depressive symptoms (n = 103)	Women without depressive symptoms (n = 242)	*P*
Age, mean (SD) (y)	35.0 (7.7)	33.9 (7.5)	35.5 (7.7)	0.07
Age at diagnosis, median (IQR) (y)	32.0 (24.0-36.0)	29.0 (23.0-35.0)	33.0 (25.0-37.0)	0.02
Years since diagnosis, median (IQR)	2.5 (1.0-7.7)	2.5 (1.1-8.2)	2.5 (1.0-7.4)	0.85
BMI, mean (SD) (kg/m^2^)	24.6 (4.8)	25.2 (5.5)	24.4 (4.4)	0.18
POI diagnoses cause				0.05
Idiopathic, n (%)	242 (70.1)	78 (75.7)	164 (67.8)	
Iatrogenic, n (%)	75 (21.7)	23 (22.3)	52 (21.5)	
Genetic, n (%)	24 (7.0)	2 (1.9)	22 (9.1)	
Autoimmune, n (%)	4 (1.2)	0	4 (1.7)	
Country of birth				0.16
The Netherlands, n (%)	282 (82.5)	80 (77.7)	202 (84.5)	
Other, n (%)	60 (17.5)	23 (22.3)	37 (15.5)	
Missing, n (%)	3 (0.9)	0	3 (1.2)	
Relationship status				0.19
Partner, n (%)	224 (64.9)	63 (61.2)	161 (66.5)	
No partner, n (%)	70 (20.3)	27 (26.2)	43 (17.8)	
Missing, n (%)	51 (14.8)	13 (12.6)	38 (15.7)	
Wish to conceive				0.27
Yes, n (%)	120 (38.3)	40 (38.8)	80 (33.1)	
No, n (%)	193 (55.9)	57 (55.3)	136 (56.2)	
Missing, n (%)	32 (9.3)	6 (5.8)	26 (10.7)	
Parity				0.08
Nulliparous, n (%)	198 (59.3)	63 (61.2)	135 (55.8)	
≥1, n (%)	136 (39.4)	40 (38.8)	96 (39.7)	
Missing, n (%)	11 (3.2)	0	11 (4.5)	
History of depression, n (%)*a*	9 (2.6)	7 (6.8)	2 (0.8)	0.001
Use of antidepressants, n (%)	31 (9.0)	19 (18.4)	12 (5.0)	<0.001
EPT use during intake, n (%)	146 (42.3)	43 (41.7)	103 (42.6)	0.89
Use of oral HT, n (%)	103 (29.9)	27 (26.2)	76 (31.4)	0.33
Use of dermal HT, n (%)	43 (12.5)	16 (15.5)	27 (11.2)	0.26
Number of days EPT use before intake, median (IQR)	42.5 (0-785.8)	71 (0-672)	18 (0-974)	0.88
OCP use during intake, n (%)	44 (12.8)	12 (11.7)	32 (13.2)	0.69
Hormone levels				
TSH mU/L, median (IQR)	1.63 (1.17-2.26)	1.50 (1.10-2.01)	1.65 (1.23-2.42)	0.04
LH IU/L, median (IQR)	26.5 (9.8-40.5)	27.8 (11.8-46.0)	25.8 (9.4-38.3)	0.16
FSH IU/L, median (IQR)	50.2 (18.6-79.9)	53.1 (24.7-84.4)	47.9 (15.6-78.6)	0.28
Estradiol, pmol/L, median (IQR)	76.0 (<55-238.5)	70 (<55-200)	79 (<55-252)	0.33
Progesterone, nmol/L, median (IQR)	<0.3 (<0.3-<0.3)	<0.3 (<0.3-<0.3)	<0.3 (<0.3-<0.3)	0.60
AMH µg/L, median (IQR)	<0.01 (<0.01-0.03)	<0.01 (<0.01-0.02)	<0.01 (<0.01-0.03)	0.46
Vitamin D, nmol/L, mean (SD)	66.5 (26.6)	66.5 (51.0-78.0)	66.0 (51.0-83.0)	0.46
Total GCS score, mean (SD)	20.7 (11.0)	29.6 (9.6)	16.9 (9.2)	<0.001
Total FertiQoL score, mean (SD)	64.0 (15.5)	53.8 (16.4)	69.4 (11.8)	<0.001
Total PROMIS score for emotional support, median (IQR)	18.0 (15.5-20.0)	16.0 (13.0-19.0)	19.0 (16.0-20.0)	<0.001
Total BDI-II score, mean (SD)	15.4 (10.0)	27.6 (7.2)	10.1 (5.3)	<0.001

Values of baseline characteristics are displayed as mean with SD, median with interquartile range (IQR) or numbers with percentages (%), depending on the distribution.

AMH, anti-Müllerian hormone; BDI-II, Beck Depression Inventory-II; BMI, body mass index; EPT, estrogen plus progestogen therapy; FertiQoL, Fertility Quality of Life; FSH, follicle-stimulating hormone; GCS, Greene climacteric scale; HT, hormone therapy; LH, luteinizing hormone; OCP, oral contraceptive pill; POI, premature ovarian insufficiency; PROMIS, Patient-Reported Outcomes Measurement Information System; TSH, thyroid-stimulating hormone; y, years.

^*a*^ Five women who reported a history of depression were still using antidepressants (all in the group of women with depressive symptoms).

Women with depressive symptoms were younger at the time of diagnosis (29.0 vs. 33.0, *P*=0.02), the distribution of POI diagnosis cause was borderline significantly different (*P*=0.05), and women with depressive symptoms had a significantly lower TSH (1.50 vs. 1.65 mU/L, *P*=0.04). Furthermore, women with depressive symptoms had significantly higher GCS scores, lower fertiQoL scores and lower total PROMIS score for emotional support (*P*<0.001). Furthermore, age, BMI, and relationship status were not significantly different between the 2 groups. In total, 35 women had a score indicative of severe depression (BDI >30), of these women 4 reported havinga history of depression and 8 were using antidepressants.

### Determinants of depressive symptoms (BDI-II >19)

To explore variables associated with depressive symptoms among women with POI, we first conducted an univariate analysis. In the univariate models (Table 2), age (OR=0.97, *P*=0.07), age at diagnosis (OR=0.97, *P*=0.04), BMI (OR=1.04, *P*=0.14), POI diagnosis cause genetic (OR=0.19, *P*=0.03), country of birth as a proxy for ethnicity (OR=1.57, *P*=0.13), relationship status (OR=0.62, *P*=0.10), TSH (OR=0.80, *P*=0.09), LH (OR=1.01, *P*=0.14), anti-Müllerian hormone (OR=0.12, *P*=0.16), total GCS score (OR=1.14, *P*<0.001), total FertiQoL score (OR=0.92, *P*<0.001), and total PROMIS score for emotional support (OR=0.82, *P*<0.001) had a *P*-value <0.20 and were therefore included in the multivariable model. The multivariable logistic regression model showed that more severe menopausal symptoms (GCS score, OR=1.13, *P*<0.001), lower emotional support (PROMIS score, OR=0.86, *P*<0.001) and younger age at diagnosis (OR=0.95, *P*=0.01) were significantly associated with higher odds of reporting depressive symptoms. POI caused by known genetic variants (OR=0.10, *P*=0.04) was associated with a lower odds of reporting depressive symptoms. This multivariable logistic regression model explained almost half of the variance in depressive symptoms (*R* ^2^=0.45).

**TABLE 2:** Univariate model: determinants of depressive symptoms (BDI-II >19)

Determinants	OR (95% CI)	*P*
Age	0.97 (0.94-1.00)	0.07*d*
Age at diagnosis	0.97 (0.94-1.00)	0.04*d*
Years since diagnosis	1.01 (0.97-1.05)	0.76
BMI	1.04 (0.99-1.09)	0.14*d*
POI diagnoses cause—iatrogenic	0.93 (0.53-1.63)	0.80
POI diagnoses cause—genetic	0.19 (0.04-0.83)	0.03*d*
POI diagnoses cause—autoimmune	0.00	0.99
Country of birth*a*	1.57 (0.88-2.81)	0.13*d*
Relationship status*b*	0.62 (0.36-1.09)	0.10*d*
Wish to conceive*c*	1.19 (0.73-1.95)	0.48
Parity	1.12 (0.70-1.80)	0.64
EPT use during intake	0.97 (0.61-1.54)	0.89
Use of oral HT	0.78 (0.46-1.30)	0.34
Use of dermal HT	1.46 (0.75-2.85)	0.26
Number of days EPT use before intake	1.00 (1.00-1.00)	0.35
OCP use during intake	0.87 (0.43-1.76)	0.69
TSH	0.80 (0.62-1.04)	0.09*d*
LH	1.01 (1.00-1.02)	0.14*d*
FSH	1.00 (1.00-1.01)	0.28
Estradiol	1.00 (1.00-1.00)	0.22
Progesterone	1.01 (0.97-1.05)	0.71
AMH	0.12 (0.01-2.31)	0.16*d*
Vitamin D	1.00 (0.99-1.01)	0.46
Total GCS score	1.14 (1.10-1.17)	<0.001*d*
Total FertiQoL score	0.92 (0.90-0.95)	<0.001*d*
Total PROMIS score for emotional support	0.82 (0.76-0.88)	<0.001*d*

Values are displayed in odds ratios (OR) with 95% confidence interval (CI).

AMH, anti-Müllerian Hormone; BDI-II, Beck Depression Inventory-II; BMI, body mass index; EPT, estrogen plus progestogen therapy; FertiQoL, Fertility Quality of Life; FSH, follicle-stimulating hormone; GCS, Greene climacteric scale; HT, hormone therapy; LH, luteinizing hormone; OCP, oral contraceptive pill; POI, premature ovarian insufficiency; PROMIS, Patient-Reported Outcomes Measurement Information System; TSH, thyroid-stimulating hormone.

^*a*^ Reference is “The Netherlands.”

^*b*^ Reference is “No partner.”

^*c*^ Reference is “No wish to conceive.”

^*d*^ *P*-value <0.20 and therefore included in the multivariate model.

### Sub-analysis

In a subgroup of women who filled in the FertiQoL (this questionnaire is only filled in when women feel grief related to infertility), the univariate model identified total GCS score, total PROMIS score for social support, total FertiQoL score, POI diagnose cause, LH, FSH, estradiol, TSH, partner status, years since diagnosis, age at diagnosis, BMI and use of dermal HT were significantly associated with depressive symptoms. The multivariable logistic regression model showed that a worse GCS score (OR=1.09, *P*<0.001), a worse FertiQoL score (OR=0.93, *P*<0.001) and the use of dermal HT (OR=3.00, *P*=0.05) were significantly associated with higher odds of reporting depressive symptoms (Table 3). This multivariable logistic regression model explained 46% of the variance in depressive symptoms (*R* ^2^=0.46).

**TABLE 3:** Multivariate model: determinants of depressive symptoms (BDI-II >19)

	OR (95% CI)	*P*
Determinants		
** **Total GCS score	1.13 (1.09-1.17)	<0.001
** **Total PROMIS score for emotional support	0.86 (0.79-0.94)	<0.001
** **POI diagnoses cause—iatrogenic	0.98 (0.47-2.04)	0.95
** **POI diagnoses cause—genetic	0.10 (0.01-0.95)	0.04
** **POI diagnoses cause—autoimmune	0.00	0.99
** **Age at diagnosis	0.95 (0.91-0.99)	0.01
Sub-analysis FertiQoL filled in (n = 212)		
** **Determinants		
** **Total GCS score	1.09 (1.04-1.13)	<0.001
** **Total FertiQoL score	0.93 (0.90-0.96)	<0.001
** **Use of dermal HT	3.00 (1.01-8.96)	0.05
Sub-analysis in women with idiopathic POI (n = 242)		
** **Total GCS score	1.13 (1.09-1.17)	<0.001
** **Total PROMIS score for emotional support	0.85 (0.77-0.94)	0.002
Sub-analysis in women aged <30 y (n = 86)		
** **Total GCS score	1.18 (1.08-1.28)	<0.001
** **Total PROMIS score for emotional support	0.81 (0.67-0.98)	0.03
Sub-analysis in women aged >30 y (n = 239)		
** **Total GCS score	1.12 (1.08-1.16)	<0.001
** **Total PROMIS score for emotional support	0.86 (0.77-0.95)	0.003
** **Age at diagnosis	0.93 (0.87-0.98)	0.01
** **TSH	0.71 (0.47-1.07)	0.10

Values are displayed as odds ratio (OR) with 95% CI.

BDI-II, Beck Depression Inventory-II; FertiQoL, Fertility Quality of Life; GCS, Greene Climacteric Scale; HT, hormone therapy; POI, premature ovarian insufficiency; PROMIS, Patient-Reported Outcomes Measurement Information System; TSH, thyroid-stimulating hormone; y, years.

In women with idiopathic POI, similar variables were identified in the univariate model. In the multivariable logistic regression model, more severe menopausal symptoms (GCS score; OR=1.13, *P*<0.001) and lower emotional support (PROMIS; OR=0.85, *P*=0.002) were significantly associated with higher odds of reporting depressive symptoms (Table 3). This multivariable logistic regression model explained 42% of the variance in depressive symptoms (*R* ^2^= 0.42).

Among women younger than 30 years, more severe menopausal symptoms (GCS scores) and lower emotional support (PROMIS scores) were significantly associated with higher odds of reporting depressive symptoms. In women older than 30 years, a younger age at diagnosis and lower TSH levels were also associated with depressive symptoms (Table 3). The full model explained 54% of the variance in depressive symptoms in women under 30 and 41% in women over 30.

Finally, we investigated which domains and specific questions in the GCS, FertiQol, and PROMIS for emotional support were significantly associated with depressive symptoms (Supplemental Tables 1–3, Supplemental Digital Content 1, http://links.lww.com/MENO/B399). Of the GCS, higher scores in the anxiety and depression domains were associated with a significantly higher odds of reporting depressive symptoms. The questions regarding panic attacks and/or anxiety, fatigue and listlessness, feeling depressed or not happy, tingling or numbness in the skin and libido loss were most significant in the multivariable logistic regression model associated with depressive symptoms. We also performed a sensitivity analysis by excluding the depression domain and the psychological domain (depression and anxiety) of the GCS and found for both scores the same significant association with depressive symptoms (*P*<0.001). In the FertiQoL, worse scores in the domains of mind and body, relation and social were associated with a higher odds of reporting depressive symptoms. The questions regarding quality of life and talking about infertility with the partner were most significant in the multivariable logistic regression model associated with depressive symptoms. In the PROMIS for emotional support, a lower score on the statement “I have someone who makes me feel appreciated” was associated with depressive symptoms.

## DISCUSSION

In this study, we have investigated the key variables associated with depressive symptoms in women with POI. The findings reveal several significant associations between depressive symptoms and various clinical and patient-reported factors. One-third of the women with POI suffered from depressive symptoms. This highlights the significant impact of depressive symptoms in women with POI, underscoring the need for health care providers to prioritize this aspect of care. An increase in the severity of menopausal symptoms, a lack of emotional support, and younger age at the time of POI diagnosis were independently related to depressive symptoms. Interestingly, a genetic cause for POI was associated with lower depressive symptoms. Subgroup analysis showed that among women experiencing fertility-related grief, lower fertility–related quality of life and limited emotional support were related to depressive symptoms.

This study is the first to investigate variables that are associated with depressive symptoms in a large cohort of 345 women with POI. While previous research on POI has explored associations between women with and without depressive symptoms, our findings contribute novel insights. Consistent with our results, women with a history of depression have been shown to experience a more severe burden of menopausal symptoms, particularly in the psychosocial and sexual domains.^13^ Our findings and those of previous studies highlight the complex interplay between menopausal symptoms, sleep disturbances, anxiety, and depression in women experiencing menopausal complaints. Research in breast cancer survivors has shown that vasomotor symptoms can disrupt sleep, leading to increased anxiety, which in turn, is a primary predictor of depression.^14^ While sleep disturbances were not directly associated with depression in this population, their strong correlation with anxiety and depression in women with POI^15^ suggests a potential indirect effect.

Unexpectedly, although a higher burden of menopausal symptoms was independently associated with depressive symptoms, vasomotor symptoms specifically (night sweats and flushes) were not, suggesting they may not be the key contributors to this association. This is consistent with the finding that hormone therapy did not significantly impact depressive symptoms. But this contradicts previous research in women with a normal menopausal age, which has demonstrated a positive association between vasomotor symptoms and depression.^16^ One potential explanation for this discrepancy lies in the distinct characteristics of POI. Compared with natural menopause, POI occurs at a younger age and often involves a more pronounced psychological impact, possibly due to the concept of “biographical disruption.” Biographical disruption refers to the significant disruption of an individual’s life trajectory and self-identity.^17^ In the context of POI, this disruption could encompass altered life goals, loss of sense of control, social stigma, and disrupted social roles. Notably, a recent study suggests that the impact of POI on daily life can be characterized by this concept.^17^

It is plausible that other domains of the menopausal experience, such as psychosocial challenges or reproductive loss, may play a more prominent role than vasomotor complaints in the association with depressive symptoms in women with POI. While the psychological impact of infertility is well-established,^18^ there is limited research on how infertility affects depressive symptoms in women with POI. A study in young breast cancer survivors demonstrated that concerns about premature menopause, menopausal symptoms, and infertility are associated with depressive symptoms,^19^ highlighting the potential importance of this loss of fertility.

Our findings underline the need for comprehensive assessment and management of menopausal symptoms, including psychosocial and sexual well-being, in women with POI. Addressing the psychological impact of POI, including the loss of fertility, is probably crucial in preventing or mitigating depressive symptoms. In addition, lower levels of social support were independently associated with depressive symptoms, which can be helpful for early detection and preventive strategies. Finally, mental health professionals should explore the specific role of fertility loss in the relationship with depression in women with POI.

Our finding that genetic causes for POI are associated with a lower risk of depression warrants further investigation to understand the underlying mechanisms. One potential explanation could be that women with a genetically linked POI diagnosis might have received more information and support due to a family history of the condition. This could lead to better emotional preparedness and coping mechanisms compared with women with a nongenetic cause of POI. However, the time between POI diagnosis and study intake was not significantly associated with depressive symptoms, suggesting that the length of time living with the diagnosis may not be strongly related to depressive symptoms in this cross-sectional sample.

However, some limitations are important to acknowledge. The observational design of the study prevents definitive conclusions about causal relationships between the identified variables and depression. Given the large number of statistical tests conducted, the likelihood of false positive findings may be increased. Therefore, the results should be interpreted with caution. We are fully aware of the statistical implications of conducting multiple comparisons, and we emphasize that the findings should be considered hypothesis-generating rather than confirmatory. We highlight the need for future studies to test these associations using predefined hypotheses and appropriate methods to adjust for multiple comparisons. Another limitation of the study is that the assessment of depressive symptoms, menopausal symptoms, and social support was conducted at a single time point at intake in women with POI, which prevents analysis of changes over time and limits our ability to identify individuals who may be at increased risk of developing depressive symptoms. However, we aim to collect additional data in the coming years, including follow-up assessments, to enable longitudinal analyses that may help clarify trajectories and support future risk prediction. Furthermore, an age-matched premenopausal control group could help delineate the independent and interactive effects of POI diagnosis, chronic condition status, and psychosocial variables on observed depressive symptoms. In addition, the study population was recruited from a single center, which may limit the generalizability of our findings to a broader population, although women with POI from all over the country attended our center of expertise. Finally, the questionnaires used may have overlapping domains, potentially inflating the observed association between some measures. We identified significant differences in other domains and a sensitivity analysis showed similar results when we excluded the “depression” and “psychological” domains from the GCS total score. Future studies may benefit from including a wider range of variables beyond those captured by the current questionnaires.

## CONCLUSION

This study provides valuable insights into the complex factors associated with depressive symptoms in women with POI. Our findings underscore the high prevalence of depressive symptoms in this population and highlight the importance of comprehensive care addressing both physical and psychological aspects of menopause at an early age. By identifying key variables such as fertility-related grief, more severe menopausal symptoms, lack of emotional support, and younger age at POI diagnosis, which might enhance a deeper understanding of this issue. While the unexpected finding that vasomotor symptoms were not the primary driver challenges previous research, it emphasizes the need for further exploration of the unique psychological impact of POI. The observed association between social support and lower depressive symptoms, as well as between fertility-related grief and depressive symptoms, suggest potential areas for future research. To fully address the mental health needs of women with POI, a multidisciplinary approach is essential, incorporating psychological support, symptom management strategies, and tailored interventions to address the specific challenges faced by this population.

## Supplementary Material
