# Supplementary material for: Depressive symptoms in women with premature ovarian insufficiency (POI): a cross-sectional observational study
Source: Menopause. 2025 Jul 15;32(12):1129–35. doi: 10.1097/GME.0000000000002614 (PMC12688449; doi:10.1097/GME.0000000000002614)
Supplement: Supplementary file 1 [file gme-32-1129-s001.docx]

**Supplemental tables**

**Index**

**Supplemental table 1.** Specific domains and questions of the GCS that are associated with depressive symptoms in a multivariate model

**Supplemental table 2.** Specific domains and questions of the FertiQoL that are associated with depressive symptoms in a multivariate model

**Supplemental table 3.** Specific questions of the PROMIS for social support that are associated with depressive symptoms in a multivariate model

**Supplemental table 1.** Specific domains and questions of the GCS that are associated with depressive symptoms in a multivariate model

| **Model 1: Domains of GCS** | | |
| --- | --- | --- |
| **Determinants^a^** | **OR (95% CI)** | **p-value** |
| Anxiety | 1.17 (1.05 – 1.29) | <0.01 |
| Depression | 1.46 (1.27 – 1.67) | <0.001 |
| Vasomotor | 0.86 (0.72 – 1.02) | 0.09 |
| Sex | 1.31 (0.99 – 1.74) | 0.06 |
| **Model 2: Questions of GCS** | | |
| **Determinants^b^** | **OR (95% CI)** | **p-value** |
| Panic attacks and/or anxiety | 1.87 (1.29 – 2.72) | <0.001 |
| Fatigue and listlessness | 1.54 (1.08 – 2.21) | 0.02 |
| Feeling depressed or not happy | 2.39 (1.61 – 3.55) | <0.001 |
| Tingling or numbness in the skin | 1.55 (1.06 – 2.26) | 0.02 |
| Libido loss | 1.41 (1.06 – 1.87) | 0.02 |

Values are displayed as odds ratio (OR) with 95% confidence interval (CI). GCS: Greene Climacteric Scale. ^a^ The results of the multivariate model only includes the domains of the GCS that were significantly associated with the outcome depressive symptoms, the domain ‘Physical’ was remove in the second step of the model (OR 1.04 [0.94 – 1.14]). ^b^ The results of the multivariate model only includes the questions of the GCS that were significantly associated with the outcome depressive symptoms, the other 16 questions did not remain significant in the final model.

**Supplemental table 2.** Specific domains and questions of the FertiQoL that are associated with depressive symptoms in a multivariate model

| **Model 1: Domains of FertiQoL (n = 167)** | | |
| --- | --- | --- |
| **Determinants^a^** | **OR (95% CI)** | **p-value** |
| Mind and body | 0.98 (0.96 – 1.00) | 0.04 |
| Relational | 0.97 (0.95 – 0.99) | <0.01 |
| Social | 0.97 (0.94 – 0.99) | 0.01 |
| **Model 2: Questions of FertiQoL** | | |
| **Determinants^b^** | **OR (95% CI)** | **p-value** |
| How is your health according to you? | 0.56 (0.30 – 1.05) | 0.07 |
| Are you satisfied with the quality of your life? | 0.28 (0.14 – 0.54) | <0.001 |
| Are you satisfied with the support you receive from friends regarding your fertility problem? | 0.69 (0.44 – 1.08) | 0.11 |
| Are you satisfied with your sexual relationship despite having fertility problems? | 0.68 (0.45 – 1.03) | 0.07 |
| Do you find it difficult to talk to your partner about your feelings about infertility? | 0.46 (0.29 – 0.72) | <0.001 |
| Are you satisfied with your relationship despite the fact that you have fertility problems? | 0.64 (0.39 – 1.05) | 0.07 |

Values are displayed as odds ratio (OR) with 95% confidence interval (CI). FertiQoL: Fertility Quality of Life. ^a^ The results of the multivariate model only includes the domains of the FertiQol that were significantly associated with the outcome depressive symptoms, the domain ‘Emotional’ was remove in the second step of the model (OR 0.99 [0.96 – 1.01]). ^b^ The results of the multivariate model only includes the questions of the FertiQoL that were significantly associated with the outcome depressive symptoms, the other 30 questions did not remain significant in the final model.

**Supplemental table 3.** Specific questions of the PROMIS for social support that are associated with depressive symptoms in a multivariate model

| **Model 1: Questions of PROMIS for social support** | | |
| --- | --- | --- |
| **Determinants^a^** | **OR (95% CI)** | **p-value** |
| I have someone who makes me feel appreciated | 0.47 (0.36 – 0.61) | <0.001 |

Values are displayed as odds ratio (OR) with 95% confidence interval (CI). PROMIS: Patient-Reported Outcomes Measurement Information System. ^a^ The results of the multivariate model only includes the questions of the PROMIS, that were significantly associated with the outcome depressive symptoms, the other 3 questions did not remain significant in the final model.
